# Cognitive bias modification for energy drink cues

**DOI:** 10.1371/journal.pone.0226387

**Published:** 2019-12-16

**Authors:** Eva Kemps, Marika Tiggemann, Mikaela Cibich, Aleksandra Cabala

**Affiliations:** School of Psychology, Flinders University, Adelaide, Australia; University of Portsmouth, UNITED KINGDOM

## Abstract

Energy drink consumption is increasing worldwide, especially among young adults, and has been associated with physical and mental health problems. In two experiments, we tested the prediction that energy drink consumption is in part driven by biased cognitive processing (attentional and approach biases), with a view to modifying these to reduce consumption. Young adults (18–25 years) who regularly consume energy drinks completed the dot probe (Exp.1; N = 116) or approach-avoidance task (Exp.2; N = 110) to measure attentional and approach bias for energy drink cues, respectively. They then underwent a cognitive bias modification protocol where they were trained to direct their attention away from pictures of energy drink cans (Exp.1), or to push a joystick away from themselves in response to these pictures (Exp.2). Following a post-training assessment of attentional (Exp.1) or approach bias (Exp.2), energy drink consumption was measured by an ostensible taste test. Regular energy drink consumers showed both an attentional and an approach bias for energy drink cues. Cognitive bias modification successfully reduced both biases. However, neither attentional nor approach bias modification significantly reduced energy drink intake. The results lend some support to incentive sensitisation theory which emphasises the role of biased decision-making processes related to addictive behaviours.

## Introduction

Energy drinks are non-alcoholic beverages that contain high levels of caffeine and other stimulants, such as taurine, guarana and ginseng. Popular brands include Red Bull, Mother and V. Since 2006 energy drink consumption has more than doubled, with global consumption amounting to 11.5 billion litres per year [1]. The largest segment of consumers are young adults, who account for about two thirds of the energy drinks market [2]. Their main reasons for consuming energy drinks are to increase alertness, combat fatigue, improve academic or sport performance, and mix with alcohol at parties [3, 4].

Despite evidence that consuming energy drinks can improve physical endurance [5] and cognitive performance [6], energy drink consumption has more often been associated with a host of negative physical and mental health consequences. Reported adverse effects include symptoms ranging in severity from headaches to heart palpitations, renal failure, seizures, and in rare cases death [7, 8]. A recent systematic review concluded that energy drink use is positively associated with a range of mental health outcomes, in particular anxiety and depression, as well as stress, PTSD and substance abuse [9]. In addition, because caffeine has similar properties to other drugs, energy drinks can be addictive. While the occasional energy drink is not problematic, it has been reported that some individuals consume four or more energy drinks per day [4]. Such excessive intake can lead to the development of tolerance and serious withdrawal symptoms upon cessation [10].

These negative health outcomes have led to a recognised need to reduce energy drink consumption. Efforts have focused on introducing tighter regulations, such as limiting the caffeine content in energy drinks, providing cautionary information on energy drink labels and restricting energy drink advertising [11]. Recommendations have also been put forward to limit the access and availability of energy drinks [12]. Energy drinks are ubiquitous in our contemporary environment; they are available and accessible from shops, petrol stations and vending machines 24/7. More recently there have been calls to impose a tax on energy drinks [8] as happens for alcohol and tobacco, and has been introduced for sugar sweetened beverages in some countries (e.g., Mexico). However, banning, restricting or taxing energy drinks does not address the underlying mechanisms that drive their consumption; nor does it empower people to regulate their own intake. The present study focused on one possible mechanism underlying energy drink consumption, namely biased decision-making processes.

An influential theory of addiction that emphasises biased decision-making as a key driver of consumption is incentive sensitisation theory [13, 14]. According to this theory, appetitive cues in the environment acquire motivational properties, or incentive salience, through a process of classical conditioning (repeated association between the cue and intake of the substance). As a result, appetitive stimuli come to be perceived as attractive and ‘wanted’. Consequently, they capture attention (attentional bias), and elicit an action tendency to approach (approach bias) and consume the substance. The activation of these processes occurs automatically, and drives consumption without necessary conscious awareness. In so doing, attentional and approach biases contribute to the development and maintenance of substance use and addiction.

In support of incentive sensitisation theory, attentional and approach biases have been demonstrated for a range of appetitive substances, including alcohol [15, 16], tobacco [17, 18], drugs [19, 20] and chocolate [21, 22]. A handful of studies has also shown an attentional bias for caffeine. Stafford and colleagues [23–25] found an attentional bias for caffeine-related stimuli among moderate and heavy coffee drinkers. To date there have been no reports of an approach bias for caffeine. Further, as yet, attentional and approach biases for energy drinks have not been investigated.

Research has further shown that attentional and approach biases are malleable and can be manipulated through targeted training, known as cognitive bias modification. Training individuals to avoid appetitive stimuli by diverting their attention or making avoidance movements has been shown to reduce attentional and approach biases, respectively. Such cognitive bias modification effects have been reported for alcohol, tobacco and chocolate [22, 26–30]. In addition, there is some evidence that a reduction in bias can produce a corresponding reduction in consumption in terms of lower intake in laboratory-based taste tests [22, 28, 31–33]. However, many studies have not found any effect of cognitive bias modification on consumption [34–37]. This lack of robust effects of cognitive bias modification on consumption is reflected in recent reviews on attentional [38] and approach bias modification [39] for a range of appetitive stimuli.

The aim of the present study was to test the prediction derived from incentive sensitisation theory that energy drink consumption is in part driven by biased decision-making processes. In two experiments we investigated whether regular energy drink consumers exhibit attentional (Experiment 1) and approach (Experiment 2) biases for energy drink cues. As a next step we targeted these mechanisms through cognitive bias modification to determine whether they could be reduced. Finally, we examined the effect of such modification on energy drink consumption. If shown to be successful, cognitive bias modification could have important implications for reducing excessive energy drink consumption and its associated adverse physical and mental health consequences. Accordingly, the following a priori hypotheses were tested: (1) regular energy drink consumers would show attentional and approach biases for energy drink cues; (2) participants trained to avoid energy drink cues would show a decrease in attentional/approach bias, whereas participants trained to attend to/approach such cues would show an increase in bias; and (3) participants trained to avoid energy drink cues would consume less of the energy drinks in a taste test than participants trained to attend to/approach such cues.

## Experiment 1 Methods

### Participants

Participants were 116 regular energy drink consumers (69 women; 18–25 years), defined as consuming energy drinks at least once per fortnight [3]. Based on previous cognitive bias modification research in the food and eating domain (e.g. [21, 28]), we aimed to recruit at least 50 participants in each of the training conditions. To ensure we reached that target, we oversampled slightly to account for attrition during testing. Power calculations using G*Power [40] indicated that the statistical power achieved with this sample size was 0.72 for small, 0.99 for medium and 1.00 for large effects, respectively. Participants were recruited from the undergraduate student population at Flinders University via online and poster advertisements for a study on beverage preferences and drinking habits, and received course credit or an honorarium in lieu of their time and commitment.

### Design

The experiment used a 2 (training condition: attend, avoid) × 2 (time: pre-training, post-training) mixed design, with training condition the between-subjects factor and time the within-subjects factor. Participants were randomly allocated to the training conditions.

### Measures and materials

#### Dot probe task

The dot probe task [41] was used to measure and manipulate attentional bias for energy drink cues. Stimuli consisted of digital photographs of cans of energy drinks and non-caffeinated soft drinks. Non-caffeinated soft drinks were chosen for the comparison control category because they provide a realistic alternative to energy drinks (they are sweet, fizzy and are sold in cans, but they do not contain caffeine). The use of two highly similar beverage categories thus provided a methodologically rigorous test of attentional bias for energy drinks specifically. To equate perceptual characteristics, all cans were photographed in the upright position against a white background.

Two sets of stimulus pairs were constructed: critical (energy drink–soft drink) and control (soft drink–soft drink). The stimuli for the critical pairs consisted of four energy drinks (‘Red Bull’, ‘Mother’, ‘V’, and ‘Monster’) and four soft drinks (‘Sprite’, ‘Sunkist’, ‘Solo’ and ‘Lift’). These brands were chosen because they are popular, familiar and recognisable. Each energy drink was paired with each soft drink to create 16 critical pairs (energy drink–soft drink). The stimuli for the control pairs consisted of another 8 soft drinks. These were paired to create 16 unique control pairs (soft drink–soft drink). Another 12 picture pairs with no beverage related content derived from Kemps et al. [28] were used for practice trials.

The dot probe task consisted of three phases: (1) a baseline assessment of attentional bias for energy drinks (pre-training), (2) a training phase in which participants were trained to either attend to or avoid energy drinks, and (3) a post-training assessment of attentional bias for energy drinks similar to the pre-training (post-training).

In the pre-training phase, participants completed a standard dot probe task. On each trial, a fixation cross was displayed in the centre of the computer screen for 500 ms, followed by the presentation of a picture pair for 500 ms. The pictures were displayed 50 mm from either side of the central position. When the picture pair disappeared, a dot probe was presented in the location of one of the previously presented pictures. Participants identified the location of the probe as quickly as possible, by pressing the corresponding keys labelled L (‘z’) and R (‘/’) on the computer keyboard.

The task commenced with 12 practice trials, followed by 128 experimental trials. In the experimental trials, each of the 16 critical (energy drink–soft drink) and 16 control (soft drink–soft drink) picture pairs was presented four times, once for each of the picture location (left or right) × dot probe location (left or right) combinations. Thus probes replaced each of the pictures in each pair with equal frequency (50/50). Trials were presented in a new randomly chosen order for each participant.

In the training phase, participants completed a modified dot probe task. Only the 16 critical (energy drink–soft drink) picture pairs were used. These were each presented 16 times, for a total of 256 trials, with each picture appearing 8 times on each side of the screen. To combat potential boredom or fatigue, participants were given a brief break halfway through the training. Attentional bias was manipulated by varying the location of the dot probes. In the attend condition, dot probes replaced energy drink pictures on 90% of trials and soft drink pictures on 10% of trials, designed to direct attention toward energy drink cues. Conversely, in the avoid condition, dot probes replaced energy drink pictures on 10% of trials and soft drink pictures on 90% of trials, designed to direct attention away from energy drink cues. A 90–10 distribution was used, as opposed to a 100–0 one, to reduce the obviousness of the contingency [29].

In the post-training phase, participants again completed the standard dot probe task.

#### Energy drink intake

Energy drink intake was assessed by a so-called taste test, a valid measure of alcohol consumption [42] and food intake [43]. In line with established protocols [31], participants were presented with the two most popular energy drinks, Red Bull and Mother, and two popular soft drinks, Sprite and Solo. The drinks were served in cups accompanied by empty cans displaying the beverage brand logo. Each cup contained 125 ml of the designated beverage. The four drinks were presented together on a tray, with drink order counterbalanced across participants and conditions according to a 4 × 4 Latin square.

Participants tasted each drink and rated it on several dimensions (e.g., sweetness, fizziness, likeability). Participants could sample as much of each beverage as they wished, and were given 10 min. to make their ratings. The remaining quantity in each cup was recorded to calculate (subtract from 125 ml) how much they had drunk.

#### Habitual energy drink consumption

Energy drink consumption habits were assessed by a brief questionnaire. Participants reported (1) how often they consume energy drinks (daily, every few days, weekly, fortnightly), (2) the average number of energy drinks they consume in a day, (3) the maximum number of energy drinks they ever consumed on any one day, (4) their preferred energy drink brand, and (5) their main reasons for consuming energy drinks.

### Procedure

Participants were tested individually in a quiet room in the Food Laboratory in a single session of approximately 45 min. After giving written informed consent, they provided demographic information (age, gender). Participants then completed the dot probe task, followed by the energy drink intake measure. Finally, they completed the habitual energy drink consumption questionnaire. The study protocol was approved by the Flinders University Social and Behavioural Research Ethics Committee.

## Experiment 1 Results

The data set for Experiment 1 is available in supplementary information (S1 Data set).

### Sample characteristics

Most participants consumed energy drinks weekly (46%), fortnightly (28%) or every few days (21%), with a minority consuming them every day (6%). On the days on which participants did consume energy drinks, they drank on average 1.44 (*SD* = .66) cans. The maximum number of energy drinks consumed on any one day ranged from 1 to 20 (*M* = 3.67, *SD* = 2.76). The most popular brand of energy drink was Red Bull (43%), followed by Mother (28%) and V (12%). The top three reasons given for consuming energy drinks were needing energy, combatting fatigue, and to mix with alcohol at parties. Table 1 shows that the experimental groups (attend, avoid) did not differ on habitual energy drink consumption. Nor did they differ on age.

**Table 1:** Means (SDs) for number of energy drinks consumed and age in Experiment 1.

	Total sample(*n* = 116)	Attend group(*n* = 58)	Avoid group(*n* = 58)	*t*(114)	*p*
Age	20.16 (2.23)	20.10 (2.32)	20.21 (2.15)	.25	.804
No. cans per day	1.44 (.66)	1.53 (.75)	1.34 (.55)	1.55	.124
Max no. cans in a day	3.67 (2.76)	3.79 (3.44)	3.55 (1.88)	.47	.640

### Attentional bias

To determine an attentional bias for energy drink cues, we compared response times to dot probes replacing energy drink and soft drink pictures of the critical trials (energy drink–soft drink pairs) at pre-training. Incorrect trials (2.86%) and outlying response times (± 2.5 *SD* from the mean) (0.81%) were eliminated. Participants were significantly faster to respond to probes replacing energy drink pictures (*M* = 355 ms) than to probes replacing soft drink pictures (*M* = 360 ms), *t*(115) = 3.14, *p* < .01, *d* = .29, indicative of an attentional bias toward energy drinks. The extent of this bias did not correlate with self-reported frequency of energy drink consumption, *r* = .01, *p* = .924.

### Attentional bias modification

To assess the effect of the attentional training, we compared response times on critical trials at post-training with those at pre-training. For each assessment phase, an attentional bias score was calculated by subtracting the mean response times to probes that replaced energy drink pictures from the mean response times to probes that replaced soft drink pictures. A positive score indicates an attentional bias toward energy drinks and a negative score a bias away from energy drinks.

**Fig 1:**
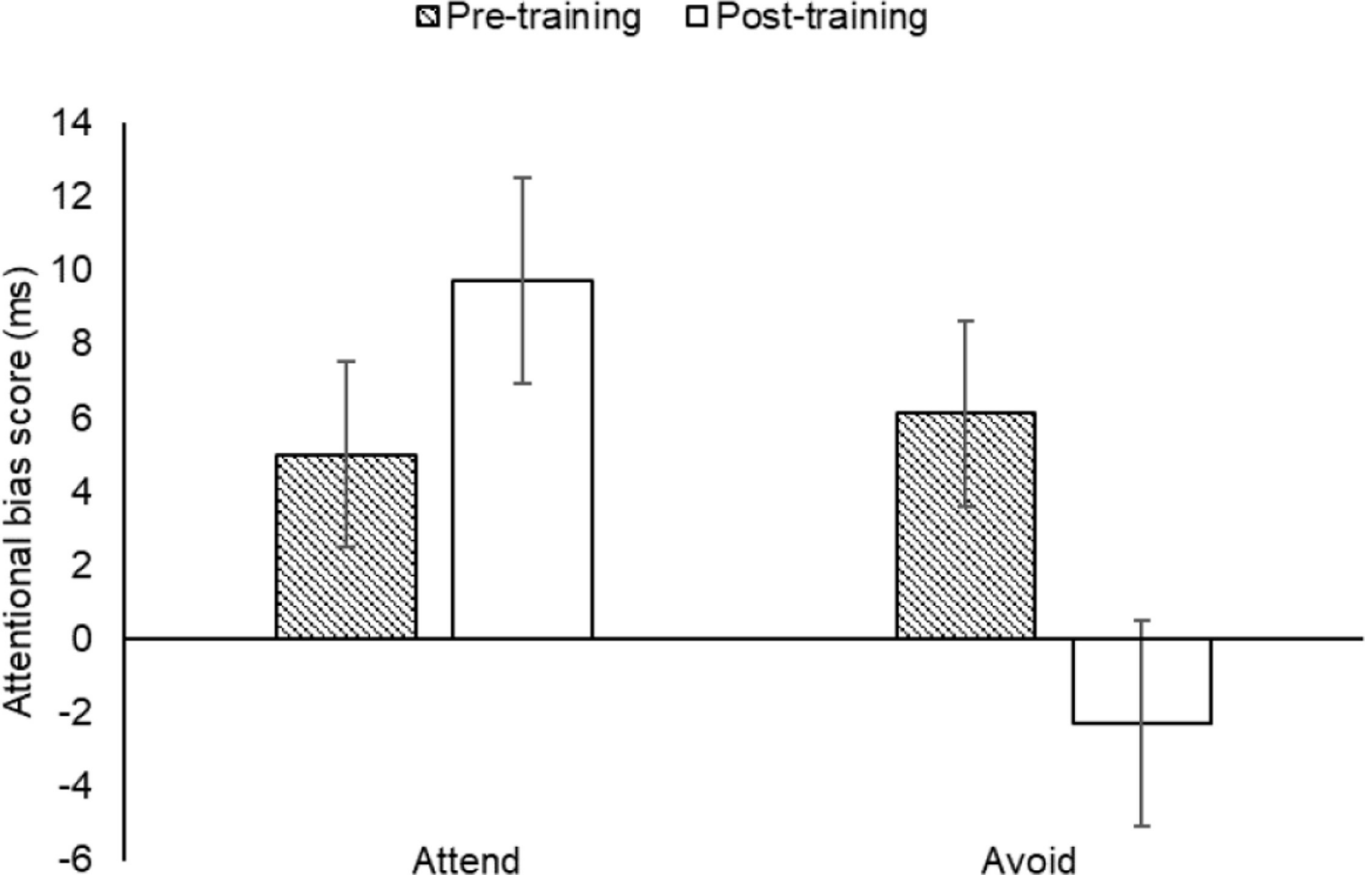
Mean attentional bias scores (with standard errors) for the training conditions (attend, avoid) at pre- and post-training in Experiment 1; * *p* < .05.

The attentional bias scores were analysed by a 2 (training condition: attend, avoid) × 2 (time: pre-training, post-training) mixed model ANOVA. There was a significant main effect of training condition, *F*(1, 114) = 3.97, *p* < .05, η_p_^2^ = .03, whereby the attend group (*M* = 5.57 ms) showed a greater attentional bias for energy drinks than the avoid group (*M* = 3.71 ms), and no significant main effect of time, *F*(1, 114) = .52, *p* = .471. Importantly, there was a significant training condition × time interaction, *F*(1, 114) = 6.47, *p* < .05, η_p_^2^ = .05. There was a significant decrease in attentional bias scores from pre- to post-training in the avoid group, *t*(57) = 2.14, *p* < .05, *d* = .28, with the attentional bias completely negated, and an increase in the attend group that was not statistically significant, *t*(57) = 1.41, *p* = .164 (Fig 1).

### Energy drink consumption

The distributions for energy drink and soft drink consumption were near-normal (skewness: ± 1; kurtosis: ± 2). A 2 (training condition: attend, avoid) × 2 (drink type: energy drink, soft drink) mixed model ANOVA examined the effect of attentional bias modification on energy drink consumption. There was a significant main effect of drink type, *F*(1, 114) = 9.75, *p* < .01, η_p_^2^ = .08, whereby participants consumed more of the energy drinks (*M* = 131 ml) than the soft drinks (*M* = 109 ml). However, there was no significant main effect of training condition, *F*(1, 114) = .49, *p* = .488, nor significant training condition × time interaction, *F*(1, 114) = .19, *p* = .664. Contrary to prediction, participants in the avoid condition consumed slightly more of the energy drinks than those in the attend condition (Table 2).

**Table 2:** Mean beverage consumption in ml (*SD* in parentheses) for the attend and avoid conditions in Experiment 1.

	Energy drinks	Soft drinks
Attend	62.58 (39.58)	53.01 (32.90)
Avoid	68.37 (44.07)	55.71 (34.57)

## Experiment 1 Discussion

The findings of Experiment 1 demonstrate for the first time an attentional bias for energy drink cues in regular energy drink consumers. In addition to demonstrating the existence of such a bias, we showed that it can also be altered. Attentional bias modification eliminated the initial attentional bias in the avoid condition where participants were trained to direct attention away from energy drink pictures. However, the observed reduction in attentional bias in the avoid group was not accompanied by a lower intake of energy drinks in the taste test.

Earlier studies in the addiction literature focused on attentional bias as one aspect of biased decision-making processes. Over recent years, the focus has shifted to approach bias. It has been argued that approach bias is likely to be a more important contributor to consumption because, unlike attentional bias, it not only has a cognitive component, but also an additional behavioural component (i.e., reaching towards the substance cues) [44]. Although approach bias has been less widely researched than attentional bias, it shows a more consistent link with consumption. A recent review of approach bias modification across several consumption domains (alcohol, cigarettes, food) concluded that approach bias modification can reduce consumption if the approach bias is successfully retrained [39]. Thus it is possible that altering approach biases for energy drink cues may be more effective than attentional bias modification in reducing energy drink consumption. This was investigated in Experiment 2.

## Experiment 2 Methods

### Participants

Participants were 110 regular energy drink consumers (76 women; 18–28 years), recruited from the undergraduate student population at Flinders University. None had taken part in Experiment 1. Power calculations indicated that the statistical power achieved with this sample size was 0.69 for small, 0.99 for medium and 1.00 for large effects, respectively.

### Design

The experiment used a 2 (training condition: approach, avoid) × 2 (time: pre-training, post-training) mixed design, with training condition the between-subjects factor and time the within-subjects factor. Participants were randomly allocated to the training conditions.

### Measures and materials

#### Approach-avoidance task

The approach-avoidance task [45] was used to measure and manipulate approach bias for energy drinks. Stimuli consisted of five pictures of energy drinks and five pictures of non-caffeinated soft drinks, displayed in cans. These included the same eight pictures that were used for the critical pairs in Experiment 1, plus one additional energy drink picture (Monster) and one additional soft drink picture (Sunkist) to increase the number of trials and thus the reliability of the data. All 10 pictures were created in both portrait (aspect ratio 3:4) and landscape (aspect ratio 4:3) format. Another 20 pictures with no beverage related content (animals) taken from Schumacher et al. [22] were used for practice trials.

In the pre- and post-training phases of the task, each trial commenced with a picture of an energy drink displayed in the centre of the computer screen. Participants responded to the format of the picture (portrait or landscape orientation), not its content (energy drink or soft drink) by pulling or pushing a joystick, thus mimicking an approach or avoidance movement, respectively. Half the participants pulled the joystick for pictures in portrait format and pushed the joystick for pictures in landscape format, and vice versa for the other half. Pulling the joystick increased the size of the picture, and pushing the joystick decreased its size, thereby enhancing the sense of approaching and avoiding, respectively. The picture disappeared once it had been pulled or pushed to its largest or smallest size, respectively. Participants were instructed to respond as quickly and as accurately as possible. Each of the 10 pictures was presented five times in each format (portrait, landscape), for a total of 100 trials. These were presented in a new random order for each participant. In the pre-training phase the experimental trials were preceded by 20 practice trials.

In the training phase, the pull-push contingencies were manipulated. In the approach training condition, 90% of the energy drink pictures were presented in pull-format and 10% in push-format (with reversed contingencies for soft drink pictures). In the avoid training condition, all contingencies were reversed, resulting in 90% push-responses to energy drink pictures and 10% pull-responses to soft drink pictures. All pictures were presented 20 times for a total of 200 trials.

### Procedure

The procedure was similar to that of Experiment 1, except that participants completed the approach-avoidance task.

## Experiment 2 Results

The data set for Experiment 2 is available in supplementary information (S2 Data set).

### Sample characteristics

As in Experiment 1, most participants consumed energy drinks fortnightly (33%), weekly (32%) or every few days (27%), with 8% consuming them every day. On the days on which participants consumed energy drinks, they drank on average 1.45 (*SD* = .58) cans. The maximum number of energy drinks consumed on any one day ranged from 1 to 6 (*M* = 2.93, *SD* = 1.40). Participants’ preferred brand of energy drink was again Red Bull (44%), followed by V (21%) and Mother (18%). The top three reasons for consuming energy drinks were combatting fatigue, needing energy and studying. As in Experiment 1, the experimental groups did not differ on habitual energy drink consumption nor age (Table 3).

**Table 3:** Means (SDs) for number of energy drinks consumed and age in Experiment 2.

	Total sample(*n* = 110)	Approach group(*n* = 55)	Avoid group(*n* = 55)	*t*(108)	*p*
Age	20.99 (2.47)	20.93 (2.46)	21.05 (2.50)	.27	.789
No. cans per day	1.44 (.66)	1.49 (.60)	1.42 (.57)	.65	.517
Max no. cans in a day	3.67 (2.76)	3.13 (1.47)	2.73 (1.31)	1.51	.135

## Approach bias

To determine an approach bias for energy drink cues, we compared response times of trials in which participants pulled the joystick in response to energy drinks pictures with those in which participants pushed the joystick in response to such pictures in the pre-training phase. Incorrect responses (3.16%) and outlying response times (± 2.5 *SD* from the mean) (2.39%) were eliminated. Participants were faster to pull (*M* = 800 ms) than to push (*M* = 819 ms) the joystick in response to energy drink pictures, *t*(109) = 2.21, *p* < .05, *d* = .21, indicative of an approach bias for energy drinks, the extent of which did not correlate with self-reported frequency of energy drink consumption, *r* = .01, *p* = .927. A similar analysis conducted on the pull (*M* = 796 ms) and push (*M* = 819 ms) responses to soft drink pictures showed an approach bias also for soft drinks, *t*(109) = 2.91, *p* < .01, *d* = .28.

### Approach bias modification

To examine the effect of the approach-avoidance training, we compared response times to energy drink pictures at post-training with those at pre-training. For each assessment phase, an approach bias score was calculated by subtracting the mean response times of trials in which participants pulled the joystick in response to energy drink pictures from the mean response times of trials in which participants pushed the joystick in response to such pictures. A positive score indicates an approach bias toward energy drinks and a negative score an avoidance bias away from energy drinks.

**Fig 2:**
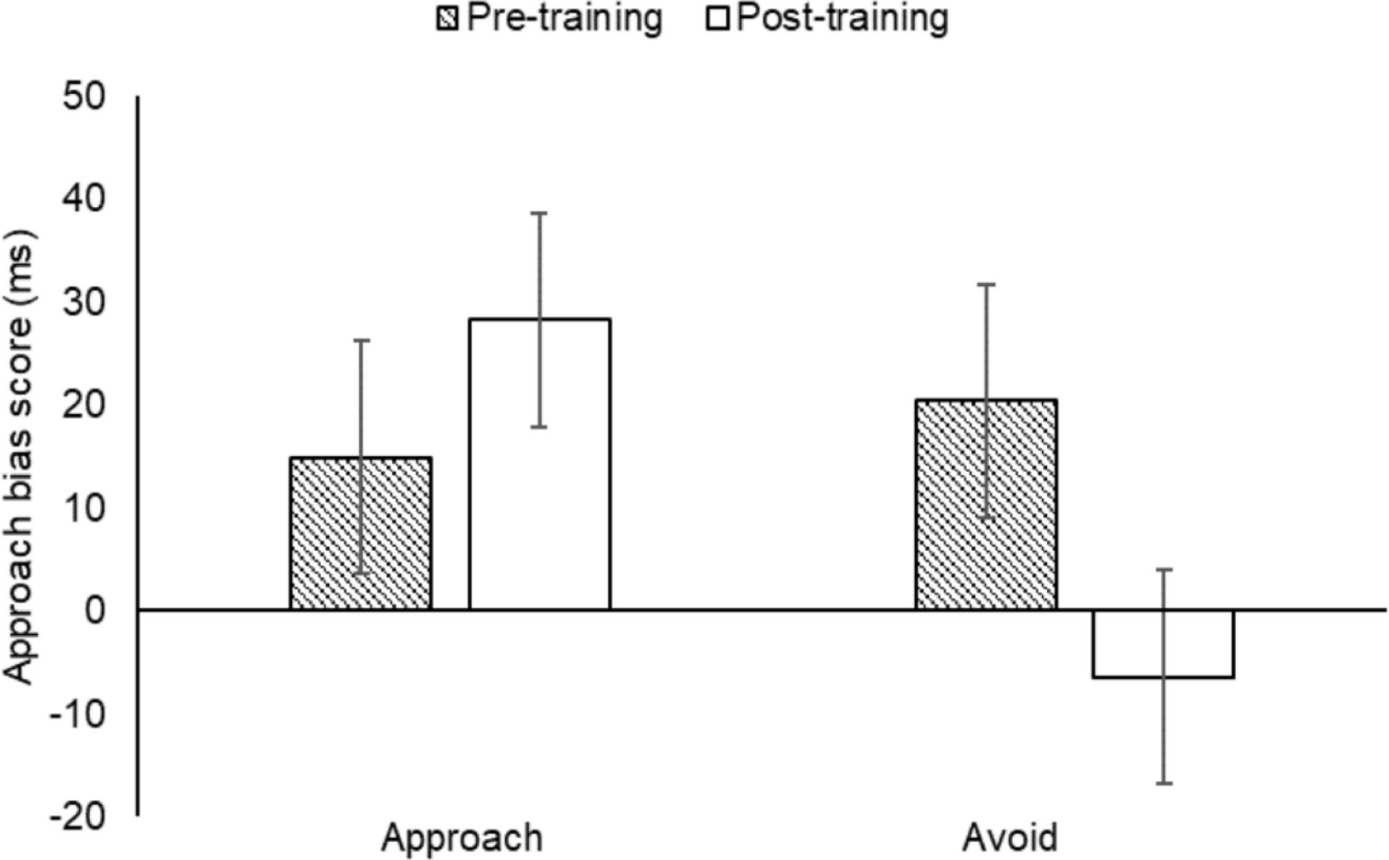
Mean approach bias scores (with standard errors) for the training conditions (approach, avoid) at pre- and post-training in Experiment 2; * *p* < .05.

The approach bias scores were analysed by a 2 (training condition: approach, avoid) × 2 (time: pre-training, post-training) mixed model ANOVA. There were no significant main effects of training condition, *F*(1, 108) = 1.30, *p* = .257, or time, *F*(1, 108) = .63, *p* = .430. However, there was a significant training condition × time interaction, *F*(1, 108) = 5.63, *p* < .05, η_p_^2^ = .05. There was a significant decrease in approach bias scores from pre- to post-training in the avoid group, *t*(54) = 2.55, *p* < .05, *d* = .35, with the approach bias completely negated, but no significant increase in the approach group, *t*(54) = 1.01, *p* = .318 (Fig 2). In contrast, a parallel analysis conducted on the approach bias scores for soft drinks showed that there were no significant main effects of training condition, *F*(1, 108) = .70, *p* = .405, or time, *F*(1, 108) = .51, *p* = .476, nor a significant training condition × time interaction, *F*(1, 108) = .05, *p* = .829.

### Energy drink consumption

Skewness and kurtosis indices indicated that energy drink and soft drink consumption were within acceptable limits of normally distributed data (skewness: ± 1; kurtosis: ± 2). A 2 (training condition: approach, avoid) × 2 (drink type: energy drink, soft drink) mixed model ANOVA investigated the effect of approach bias modification on energy drink consumption. There was no significant main effect of training condition, *F*(1, 108) = .58, *p* = .450. Unlike in Experiment 1, there was also no significant main effect of drink type, *F*(1, 108) = 2.36, *p* = .127; however, the means were in the same direction with participants consuming more of the energy drinks (*M* = 120 ml) than the soft drinks (*M* = 111 ml). Although the means indicated that participants in the avoid condition did consume slightly less of the energy drinks than participants in the approach condition, the training condition × time interaction was not statistically significant, *F*(1, 108) = .42, *p* = .518 (Table 4).

**Table 4:** Mean beverage consumption in ml (SD in parentheses) for the approach and avoid conditions in Experiment 2.

	Energy drinks	Soft drinks
Approach	63.17 (34.87)	56.79 (29.67)
Avoid	57.02 (34.41)	54.42 (33.59)

## Experiment 2 Discussion

In addition to an attentional bias, the findings of Experiment 2 demonstrated the existence of an approach bias for energy drink cues (as well as soft drink cues) in regular energy drink consumers. Furthermore, approach bias modification, like attentional bias modification, eliminated the initial approach bias in the avoid group. In line with Experiment 1, approach bias modification did not have a significant effect on energy drink consumption in the taste test. However, results were in the expected direction in that energy drink intake in the avoid group was lower than in the approach group.

## General discussion

The present study is the first to investigate biased decision-making processes as a possible factor in energy drink consumption. In two experiments, we demonstrated that regular energy consumers show both an attentional and approach bias for energy drink cues. We further showed that cognitive bias modification successfully reduced both biases. However, neither attentional nor approach bias modification had a significant effect on energy drink intake in a taste test.

The main focus of the current study was to examine biased decision-making processes as a possible contributing factor to energy drink consumption. Our finding that regular energy drink consumers show both an attentional and an approach bias for energy drink cues adds energy drinks to the list of substances for which cognitive biases have been shown, including alcohol, tobacco, drugs and chocolate [15–22]. The popularity of energy drinks is likely due to their high levels of caffeine. Although a handful of studies have shown an attentional bias for caffeine in coffee drinkers [23–25], none have as yet demonstrated an approach bias. Future studies will need to determine whether energy drink consumers show attentional and approach biases for energy drinks because of the addictive properties of caffeine.

Theoretically, the observed attentional and approach biases for energy drink cues are consistent with the propositions of incentive sensitisation theory [23, 24]. The pictures of energy drink cans used in the dot probe and approach-avoidance tasks likely would have provided cues with high incentive salience for our sample of regular energy drink consumers. These stimuli would therefore have been perceived as attractive and ‘wanted’. As a result, according to the theory, participants automatically directed their attention to the energy drink pictures in Experiment 1, and exhibited an automatic approach action tendency towards them in Experiment 2.

The observed approach bias here was not specific to energy drink cues, with participants also showing an approach bias for soft drink cues. These biases are, however, not necessarily mutually exclusive. First, research shows that individuals who consume energy drinks often also consume soft drinks [46]. Second, the nature of the approach-avoidance task makes it is possible for participants to demonstrate an approach bias for both the target and the control stimulus categories, as on each trial participants are presented with, and respond to, a single stimulus. By contrast, in the dot probe (attentional bias) task the target and control stimuli are paired and compared directly within trials.

Importantly, we found that the observed attentional and approach biases for energy drink cues are malleable. Attentional and approach bias modification eliminated the initial attentional and approach biases, respectively. It is noteworthy that these findings were specific to energy drink cues. Although the training procedures were conceptualised as targeting energy drinks, they inherently also target soft drinks (in the opposite direction). This demonstrates for the first time that biased processing of energy drink cues can be manipulated through targeted training, just as has been shown for other substances, in particular, alcohol, tobacco and chocolate [22, 26, 28–30].

Contrary to prediction, however, the observed reductions in attentional and approach bias were not accompanied by a corresponding lower intake of energy drinks, although the consumption pattern was in the expected direction following approach bias modification. These findings are at odds with some previous reports of reductions in alcohol and chocolate consumption following attentional [28, 31, 33] or approach [22, 30, 32] bias modification. However, many other studies have found no reduction in consumption following cognitive bias modification [34–37].

One possible explanation for the lack of cognitive bias modification effects on energy drink consumption is that a single training session, as used in the present experiments, may not have been sufficient to translate the reductions in bias into a reduction in consumption. A comparison of a single versus multiple attentional bias modification training sessions showed that although a single training session was sufficient to reduce attentional bias for chocolate, it did not reduce chocolate consumption; however, five training sessions of attentional bias modification did reduce chocolate consumption [47]. Multiple attentional bias modification training sessions have also been shown to reduce alcohol consumption [48]. Thus future research could determine whether more intensive cognitive bias modification training across multiple sessions would similarly bring about a reduction in energy drink consumption. This could be particularly the case for approach bias modification which, in contrast to attentional bias modification, did produce a slightly lower energy drink intake in the avoid group than in the approach group. In addition, a combination of both attentional and approach bias modification might be maximally effective in reducing energy drink consumption. Sharbanee and colleagues [32] showed that attentional and approach biases are distinct mechanisms that make independent contributions to alcohol consumption.

An alternative explanation for the lack of cognitive bias modification effects on energy drink consumption could be in the choice of consumption measure. Following previous studies, we used a well-established taste test protocol [31]. In our version, participants were presented with a limited selection of pre-poured energy drinks and required to taste and rate each one. This is a very different scenario from being asked to direct attention toward, or reach for, pictures of energy drink cans. Future research could use a consumption measure that more closely aligns with the attentional and/or approach bias tasks, for example, choosing a can of beverage from a vending machine. Such choice tasks are beginning to be used in the food domain [49, 50]. In addition, the taste test provides a one-off consumption measure in the laboratory. Future research could include follow-up measures of naturally occurring energy drink consumption.

Nevertheless, the present single session laboratory experiments provide proof of concept that attentional and approach biases for energy drink cues exist in regular energy drink consumers, and that these biases can be modified. In doing so, they provide a useful starting point for future clinical studies. If multi-session cognitive bias modification interventions can reduce energy drink intake, these could pave the way for randomised controlled trials in clinical samples such as heavy energy drink consumers who show signs of caffeine addiction (dependence, tolerance, withdrawal symptoms [10]) or experience negative physical and/or mental health consequences. Effective interventions are all the more important given the rising rates of energy drink consumption [1].

Furthermore, the reported frequency and amount of self-reported energy drink consumption in the present experiments puts some participants at risk of adverse physical and mental health outcomes associated with excessive energy drink intake [7–9]. These consumption habits in our Australian sample are similar to those of a Canadian sample of young adults [4]. In line with the latter study, and that of Malinauskas et al. [3] who surveyed energy drink consumption patterns in American college students a decade earlier, the main reasons reported for consuming energy drinks by the present sample were to increase energy, combat fatigue, study and drink with alcohol at parties. These reflect the concerns of young adults, who in Western societies are the core consumers of energy drinks and the target age group for the marketing of energy drinks [11].

The present study has several notable strengths. First, by focusing on both attentional and approach biases, it provided a more encompassing assessment of biased decision-making processes related to energy drink consumption. Second, the experiments used well-established tasks and protocols to assess and retrain these biases, and to measure energy drink intake. Third, the stimuli of the dot probe and approach-avoidance tasks were created to be perceptually uniform. All depicted cans of beverages in the upright position photographed against a white background. Finally, the control stimuli consisted of non-caffeinated soft drinks to provide a highly similar comparison category to energy drinks, and thus yield a robust test of attentional and approach biases for energy drink cues.

However, like most research, the current experiments are also subject to a number of limitations. First, the participants in both experiments were all regular energy drink consumers. Thus it has not been determined whether the observed attentional and approach biases are unique to energy drink consumers. Future research should include a comparison control group of non-consumers to establish the specificity of these biases in energy drink consumption. Second, the samples in both experiments consisted of undergraduate student volunteers who were not necessarily motivated to curb their energy drink intake. A recent chapter and review of cognitive bias modification pertaining to alcohol consumption concluded that effects on intake may be more reliably observed in individuals who are motivated to change their consumption behaviour [51, 52]. Thus future research should specifically recruit energy drink consumers who wish to reduce their intake. Third, as there is little variety in the everyday presentation of energy drinks (they are generally sold and served in a can), the same pictures were used for the assessment and training phases of the dot probe and approach-avoidance tasks. However, to increase generalisability, future research could test whether the observed changes in attentional and approach biases extend to untrained stimuli, as well as to different tasks assessing attentional and approach-avoidance tendencies, in line with some studies in the alcohol domain [29, 30].

In conclusion, the present experiments have contributed to the literature by demonstrating that regular energy drink consumers show attentional and approach biases for energy drink cues. They further showed that these biases can be successfully reduced via cognitive bias modification. However, such modification did not reduce energy drink consumption in the laboratory. Future research might determine whether more intensive training in samples who are motivated to reduce their energy drink intake could achieve better outcomes.

## Supporting information

S1 Data set Data set of Experiment 1. (SAV)

S2 Data set Data set of Experiment 2. (SAV)
